# Temporal variation in the recovery from impairment in adriamycin-induced wound healing in rats

**DOI:** 10.1186/1740-3391-5-6

**Published:** 2007-10-10

**Authors:** Haluk Alagol, Soykan Dinc, Bilgen Basgut, Nurettin Abacioglu

**Affiliations:** 1 Department of General Surgery, Ankara Oncology Training and Research Hospital, Demetevler, Ankara, Turkey; 2 Department of Pharmacology, Gazi University, 06330, Hipodrom, Ankara, Turkey

## Abstract

**Background:**

An adriamycin-induced impairment of wound healing has been demonstrated experimentally in rats. The purpose of this study is to investigate a possible temporal variation in recovery from the impairment of wound healing caused by adriamycin administration.

**Methods:**

The subjects were 120 female Spraque-Dawley rats. They were divided into eight groups, undergoing adriamycin administration (8 mg/kg, i.v.) at 9 a.m. or 9 p.m. on day 0 and laparotomy on day 0, 7, 14 or 21. Blast pressures were recorded after the incision line had been opened, and tissue samples were kept at -30°C for later measurement of hydroxyproline levels.

**Results:**

Adriamycin treatment in rats at 9 p.m. resulted in significantly lower blast pressure levels than treatment at 9 a.m. between days 7 and 21, indicating a lag effect of healing time in wounded tissues. However the decreased hydroxyproline levels were not changed at these days and sessions.

**Conclusion:**

It is concluded that adriamycin-induced impairment of wound healing in adult female rats exhibits nycthemeral variation.

## Background

Surgical operation and chemotherapy are concurrent applications in the treatment of various cancer cases. One of the disadvantages of such a concomitant treatment is retardation of healing time in the wounded tissues. The lag effect of healing time of injured tissues is due to, and interrelated with, the circadian dosing time, as dosing time influences the extent of toxicity of some 30 anticancer drugs, including cytokines and cytostatics, in mice or rats. Selection of the proper circadian dosing time maximizes efficacy and minimizes toxicity [1-3].

Adriamycin is a broad-spectrum anthracycline-derivative intercalating agent with many clinical side effects. Adriamycin-induced impairment of wound healing was first demonstrated by Devereaux et al. in rats [4,5], and many studies have been conducted since then to clarify the mechanisms involved. It has been found that the wound-healing impairment depends on direct inhibition of mitosis in local fibroblasts and keratinocytes or myelosuppression of platelets and inflammatory cells, which are important in recovery from injury [6-8]. The toxicity and efficacy of adriamycin can be modulated by the selection of optimal dosing times in a chronotherapeutic manner [9,10], as is the case for other drugs as well [11].

In a previous study, we showed that the optimal timing for surgery after adriamycin treatment in rats is before the 7th day or after the 35th day [12]. The aim of the present study is to investigate if there is a time-of-day effect on the recovery time of adriamycin-induced wound-healing impairment in rats.

## Methods

Locally-bred female Spraque-Dawley rats – weighing 250–300 g, clinically healthy, non-pregnant, and non-lactating – were used. Rats were kept in individual cages under a light-dark cycle with 12 h of light per day (100 lux illuminance, cool fluorescent bulbs, lights on from 8 a.m. to 8 p.m.). Photosafe red bulbs were used to facilitate the injections during the 12-h dark phase. In the pre- and post-operative periods, animals were given a standard commercial laboratory diet and water ad libitum. Protocols of animal husbandry and experimentation followed applicable regulations at the Gazi University Animal Housing facilities. All experiments were performed during the months of June and July to avoid the potential impact of seasonal biological rhythms on the findings.

### Experimental protocols

#### Administration of adriamycin and laparotomy

As summarized in Table 1, the initial pool of 120 rats was divided into two equal groups based on the time of adriamycin administration (8 mg/kg i.v, Adriblastina, Deva, ıstanbul, Turkey): either 9 a.m. or 9 p.m. Adriamycin was administered only once on day 0 in both groups by injection into a dorsal tail vein under ether sedation. Each group was subdivided into 4 subgroups depending on the day of laparotomy (day 0, 7, 14, or 21). Laparotomies were conducted under ketamine-xylasine anesthesia (100 mg/kg ketamine and 5 mg/kg xylasine) as a 4 cm midline abdominal incision. The abdominal layers were closed with 4/0 polypropylene matrix sutures (Prolene; Ethicon, Edinburgh, UK).

**Table 1:** Setup of experimental rat groups

	**Adriamycin (8 mg/kg i.v.)**	
	
**Laparotomy Day**	**9 a.m**	**9 p.m**
0	Group 1	Group 2
7	Group 3	Group 4
14	Group 5	Group 6
21	Group 7	Group 8

Sample size for each groups n = 15.

#### Determination of blast pressure

Blast pressure was determined one week after the laparatomy. The animals were killed by excess ether anesthesia, and the abdominal sutures were removed. An passage was opened to the vaginal apex with the help of a lancet. A balloon was inserted into the peritoneal cavity through the incision and was filled with isotonic NaCl solution infused at a constant rate of 20 ml/min. An intraluminal pressure manometer attached to the apex of the balloon measured pressure in mmHg (Bİçakçİlar, ıstanbul, Turkey). The pressure recorded at the time when the incision line opened was considered the explosion pressure.

#### Measurement of hydroxyproline levels

A full layer of the abdominal wall in the laparotomy area, 2 cm from the skin edge, was collected and kept at -30°C for the hydroxyproline evaluation. The enzyme levels were evaluated by Bergman and Loxley's method [13]. The hydroxyproline concentrations were evaluated as μg/mg in the wet tissue.

### Statistical analysis

The results are presented as the means ± standard error of the means (SEM). Since the distributions of values were normal and the variances were homogeneous, the light-dark cycle mean values (i.e., temporal variation) of the parameters were analyzed by one-way analysis of variance (ANOVA) followed by the post-hoc Tukey-Kramer multiple comparisons test. When the data did not follow a Gaussian distribution as revealed by Barlett's test, the Kruskal-Wallis test followed by the Dunns test for multiple comparisons was used. Differences between time groups were assessed by the unpaired Student's t-test when necessary. A probability level of less than 0.05 was considered to be statistically significant.

## Results and Discussion

No rat died in the course of the study. Hyperemia, due to adriamycin leakage around the dorsal line of tail vein, was observed in one rat from the 1^st ^group, two rats from the 4^th ^group, one rat from the 6^th ^group and one rat from the 7^th ^group.

The mean blast pressures in groups 2, 4, 6, 8 (9 p.m.) were significantly lower than in groups 1, 3, 5, 7 (9 a.m.) on days 0, 7, 14, and 21 (Table 2). The simultaneous decreases in hydroxyproline levels in the various groups were not statistically different (Table 3).

**Table 2:** Temporal variation induced by adriamycin (8 mg/kg, i.v.) administration on blast pressure after laparotomy

	**Blast pressures (mmHg)**	
**Laparotomy Day**	**Injection times of adriamycin**	
	
	**9 a.m**	**9 p.m**

0	112.5 ± 12.2*	101.8 ± 5.6
7	97.1 ± 4.2*	86.3 ± 6.3
14	79.1 ± 8.0*	65.1 ± 5.3
21	83.1 ± 3.7*	72.0 ± 3.1

Data represents the means ± S.E.M; Sample size for each group: n = 15.

* 9 a.m. vs 9 p.m. on same laparatomy days p ≤ 0.05

**Table 3:** Temporal variations induced by adriamycin (8 mg/kg, i.v.) administration on hydroxyproline levels after laparotomy

	**Hydroxyproline (μg/mg wet tissue)**	
**Laparotomy Day**	**Injection times of adriamycin**	
	
	**9 a.m**	**9 p.m**

0	7.0 ± 1.1	6.8 ± 0.9
7	5.7 ± 0.2	5.6 ± 0.2
14	5.9 ± 0.9	5.8 ± 0.9
21	5.7 ± 0.3	5.7 ± 0.4

Shown are means ± S.E.M. Sample size for each group n = 15. * 9 a.m. vs 9 p.m. on same laparatomy days p ≤ 0.05

The multimodal approaches applied to improve recovery after cancer treatment has expanded the boundaries of oncological surgery. Many surgeons have to operate their patients under active chemotherapy or they have to forward their patients to adjuvant chemotherapy soon after the operation. Multiple surgical interventions are usually performed to investigate the efficiency of the debulking surgery and the efficiency of antineoplastic recovery. However, chemotherapy has negative effects on wound healing [14].

Adriamycin is a chemotherapeutic agent of the anthracycline group with wide-spectrum action. It is one of the antineoplastics most often used. However, it has many side effects. It may also be toxic to the injury under treatment if applied preoperatively. It decreases the scar collagen accumulation and so it decreases the injury tension [15]. Many studies have been conducted in order to clarify the reason for the effect of adriamycin on wound healing. It has been recently shown that the effect on wound healing depends on the myelosuppressive effect of adriamycin [16].

Our previous study showed that hydroxyproline levels were decreased significantly 7, 14, 21, and 28 days after adriamycin treatment, as compared with a control group. On the contrary, hydroxyproline levels on day 0 and day 35 after adriamycin treatment were not changed. According to these results, we suggested that the optimal timing for surgery after adriamycin treatment is after the 35th day or before the 7th day, and surgery is not recommended between the 14th and 28th days [12].

In the present study, the effect of adriamycin applied at different times was evaluated. The reason for using blast pressure was to investigate a relatively early period of the injury recovery. On the other hand, in order to answer the question as to the biological time-structure of the injury recovery, we arranged the 9 a.m. and 9 p.m. groups.

In various studies, the measurement of hydroxyproline level was used as thebiochemical parameter for injury recovery. The decrease in the level of hydroxyproline corresponds to the decrease in the amount of collagen. In many earlier studies, the anastomoses evaluation was done in the intestines, and a decrease in hydroxyproline level was found [17].

It is very natural to observe a postoperative decrease in the hydroxyproline level depending on lysis of collagen in 2–4 days. Due to the increase of the collagen synthesis, the level of hydroxyproline may also increase. Many factors such as infection, hypovolemia, prostaglandins, vitamin A, aprotonin, statins, and the nutrition conditions can affect the levels of hydroxyproline [18-23].

Total and dialyzable hydroxyproline excreted in urine is a collagen-related proliferation marker in bone, cartilage, soft tissue and skin [24]. Urinary excretion of dialysable and non-dialysable hydroxyproline varies with age in rats and exhibits diurnal fluctuations with minima and maxima appearing at the end of the dark and light fraction of the period, respectively [25]. The low hydroxyproline levels seen in many studies support the inference of a connection between deterioration of recovery from injury and decreased collagen. Thus, both enzyme levels and collagen formation must be considered in the evaluation of injury recovery [17]. Collagen-induced proliferation in wounded tissues displays enhanced mitotic activity in the relevant cells as was shown in injured adult female rat rectal epithelium with an exhibition of a diurnal variation with maximal activity during the day and minimal activity during the night [26].

Adriamycin exerts its effects by binding to nucleic acids, cell membranes and plasma proteins, and by inhibiting nucleic acid synthesis and mitotic activity. Most of these effects were suggested to impair wound healing by directly inhibiting the mitosis of wound repair cells [7]. On the other hand, the first studies in which the adriamycin was used in 1977 showed that the decrease in tumor size following recovery from adriamycin in a transplanted plasmacytoma of the rat was dependent on the application time of the medicine [27]. In other words, when the animals were subjected to adriamycin soon before they woke up at the end of their resting time, the fastest decrease in tumor size was observed. In our study, the decrease in blast pressure at night was found to be significant in all groups, but a concurrent decrease in hydroxyproline levels was not observed. We found that administration of adriamycin in the morning was more effective than administration during the night.

Extrapolation of these findings to human patients must take into consideration the fact that humans are diurnal but rats are nocturnal. Adriamycin cancer chemotherapy occurring in conjunction with therapeutic surgery must be take into consideration the optimal time for adriamycin administration with in order to increase efficacy and reduce toxicity. It is expected that in human patients adriamycin treatment will have greater efficacy and lesser toxicity when administered in the evening.

## Conclusion

Our results suggest that adriamycin-induced impairment of wound healing could be ameliorated by administration of the drug in the evening.

## Competing interests

The author(s) declare that they have no competing interest.

## Authors' contributions

HA directed the study and participated in data collection. SD participated in the design of the study. BB performed statistical analysis and coordinated the participation of the other contributors. NA directed the study, participated in data collection, and wrote the final version of the manuscript. All authors read and appoved the final manuscript.
